# Stem Cells from Human Exfoliated Deciduous Teeth Attenuate Atopic Dermatitis Symptoms in Mice through Modulating Immune Balance and Skin Barrier Function

**DOI:** 10.1155/2022/6206883

**Published:** 2022-07-21

**Authors:** Hao Xiong, Jin Yang, Tao Liu, Guangren Liu, Yongzhi Han, Xiuqin Dong

**Affiliations:** ^1^Department of Dermatology, Guangdong Provincial People's Hospital, Guangdong Academy of Medical Sciences, Guangzhou, China; ^2^Department of Allergy and Immunology, Huashan Hospital, Fudan University, Shanghai, China; ^3^Department of Dermatology, Huashan Hospital, Fudan University, Shanghai, China

## Abstract

Atopic dermatitis (AD) is a chronic skin inflammatory disease associated with immune abnormalities and disrupted skin barrier function. Mesenchymal stem cells (MSCs) have been suggested as an alternative therapeutic option in AD. Stem cells from human exfoliated deciduous teeth (SHEDs) are a unique postnatal stem cell population with high immunomodulatory properties. The aim of this study was to explore the effects of SHEDs on AD in the BALB/c mouse model induced by 2,4-dinitrochlorobenzene (DNCB). SHEDs were administrated intravenously or subcutaneously, and clinical severity, histopathological findings, skin barrier function, and organ indexes were evaluated. Skin tissue cytokine mRNA levels and serum cytokine protein levels were further analysed. SHED administration significantly alleviated AD clinical severity, including dermatitis scores, ear thickness, scratching behaviour, and infiltration of mast cells. In addition, disrupted skin barrier function and enlarged spleens were restored by SHED administration. Further, SHED treatment reduced the levels of IgE, IgG1, and thymic stromal lymphopoietin (TSLP) in the serum and the modulated expression of Th1-, Th2-, and Th17-associated cytokines in skin lesions. In conclusion, SHEDs attenuated AD-like skin lesions in mice by modulating the immune balance and skin barrier function. SHEDs could be a potential new treatment agent for AD.

## 1. Background

Atopic dermatitis (AD) is one of the most common inflammatory skin disorders and is characterised by recurrent eczematous lesions and intense pruritus [1]. AD typically develops in childhood and may persist into adulthood, showing a prevalence in 20% of children and 10% of adults in developed countries [2]. AD pathogenesis is associated with skin barrier dysfunction, skewed allergic inflammation, and skin microbiome abnormalities [3]. Conventional treatment options for AD include topical corticosteroids and calcineurin inhibitors and systemic nonspecific immunosuppressants such as azathioprine, ciclosporin, methotrexate, and phototherapy. Recently, targeted therapies involving dupilumab, omalizumab, and Janus kinase inhibitors such as baricitinib and upadacitinib have emerged [4]. These treatments have therapeutic benefits; however, their utility is limited owing to adverse events, safety concerns regarding long-term use, and high cost. Hence, the development of novel and more effective therapies for AD with fewer side effects is urgently needed.

In recent years, mesenchymal stem cells (MSCs) have emerged as a promising treatment option for several immune-mediated diseases, including asthma, allergic rhinitis, inflammatory bowel, and graft-versus-host diseases, and other inflammatory disorders [5–9]. Therapies involving different types of MSCs, such as marrow-derived mesenchymal stem cells (BM-MSCs), umbilical cord blood-derived mesenchymal stem cells (UCB-MSCs), and adipose-derived mesenchymal stem cells (AD-MSCs), have been proposed as novel strategies for the treatment of AD in animal models and humans. MSCs can exert therapeutic effects in AD through multiple ways, such as by inhibiting T-lymphocyte activation, regulating B-lymphocyte maturation, and reducing mast cell degranulation [10]. However, identifying accessible tissue sources from which MSCs can be safely obtained at a low cost and without ethical hurdles is the main challenge at present.

Stem cells from human exfoliated deciduous teeth (SHEDs) are a unique postnatal stem cell population that originates from the cranial neural crest. SHEDs are isolated from the residual pulp of exfoliated deciduous teeth in children [11] and can differentiate into various cell types, including osteogenic, chondrogenic, adipogenic, neural, endothelial cells, and odontoblasts [12]. Moreover, SHEDs express the MSC surface markers CD105, CD90, and CD73 and the early cell surface molecules CD146 and STRO-1 [13]. SHEDs have high proliferation rates, telomerase activity, cell population doubling time, and immunomodulatory ability, and they form sphere-like clusters. When compared with methods for obtaining other MSC types, obtaining SHEDs is atraumatic, with minimal ethical concerns regarding their extraction procedure and usage [12].

The immunomodulatory properties of SHEDs have been previously verified in vitro in an allergic rhinitis mouse model [13, 14]. However, only a few studies have investigated SHED application in AD treatment. In the present study, we explored the therapeutic effects of SHEDs in an AD mouse model. We analysed AD-like skin lesions, skin barrier function, histopathology, and changes in immunological balance in relation to SHED therapy. Our study provides a basis for novel SHED-based therapies for AD.

## 2. Materials and Methods

### 2.1. Animals and Materials

Eight-week-old female BALB/c mice were purchased from the Guangdong Animal Centre (Guangzhou, China) and housed under specific pathogen-free conditions at 22°C ± 2°C, 45% ± 10% relative humidity, and a 12 h light/dark cycle. Furthermore, 2,4-dinitrochlorobenzene (DNCB) was purchased from Sigma-Aldrich (St. Louis, MO, USA). Acetone and olive oil were obtained from Aladdin (Shanghai, China). Enzyme-linked immunosorbent assay (ELISA) kits were purchased from BD Biosciences (San Jose, CA, USA). The TRIzol reagent was purchased from Thermo Fisher Scientific (Waltham, MA, USA).

### 2.2. AD Model Establishment and SHED Administration

**Figure 1:**
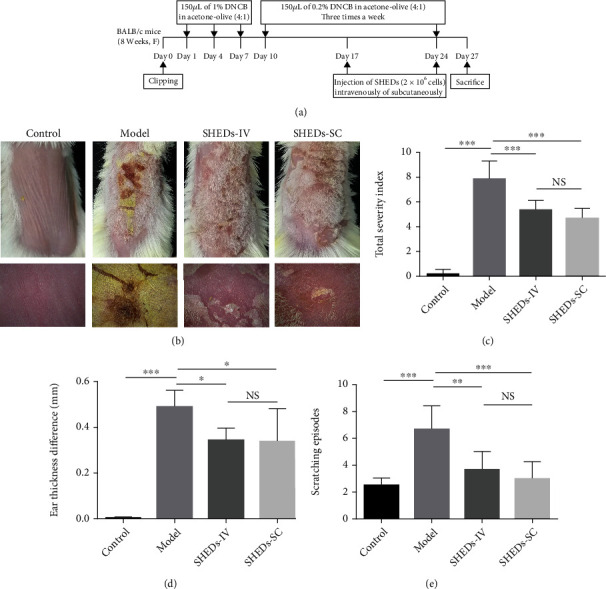
Experimental procedure and effect of SHEDs on the clinical features of AD-like symptoms induced by DNCB in BALB/c mice at the time of sacrifice. (a) Schematic diagram of the experimental procedure for the induction of AD lesions and SHED administration. (b) Cutaneous manifestations in mice in each group. (c) Clinical skin severity scores of AD-like skin lesions in BALB/c mice of each group. The total score is the sum of individual scores determined based on the symptoms of erythema/haemorrhage, scarring/dryness, edema, and excoriation/erosion. (d) Ear thickness difference in BALB/c mice of each group. (e) Scratching behaviour difference in BALB/c mice of each group. All the data are expressed as mean ± SD (*n* = 6). Three repeated times of each experiment were conducted.

After a 1-week period of acclimation to the new environment, the mice were randomly divided into four groups of six mice as follows: (1) control, (2) model, (3) model+SHED intravenous injection (SHED-IV), and (4) model+SHED subcutaneous injection (SHED-SC) groups. The AD model was established as previously described, with some modifications. As shown in Figure 1, the upper backs of the mice were shaved using a clipper. After 24 h, 150 *μ*L of 1% DNCB was dissolved in a mixture of acetone and olive oil (3 : 1) and applied to the dorsal skin and the right ear on days 1, 4, and 7. For the next three weeks, 150 *μ*L of 0.5% DNCB was applied to the same site thrice per week. The control mice were treated with acetone and olive oil (3 : 1).

SHEDs (passage 4) were provided by CAR-T (Shanghai) Biotechnology Co., Ltd. (Shanghai, China). The cells were collected from the pulp tissues of naturally exfoliated deciduous teeth of children aged 6–8 years, with informed consent. SHEDs were characterised according to the previous studies on morphology, surface markers, and differentiation capacity [15]. The administration of SHEDs referred to the previous studies with minor modifications [16–18]. SHEDs were suspended at a concentration of 2 × 10^7^ cells/mL, and 2 × 10^6^ cells were injected intravenously or subcutaneously on days 17, 24, and 31. All animals were sacrificed on day 34, and their skin tissues, internal organs, and blood samples were collected for further analysis.

### 2.3. Measurements of Skin Lesion Severity, Ear Thickness, and Scratching Episodes

Dermatitis scores of 0 (none), 1 (mild), 2 (moderate), or 3 (severe) were assigned to each of the following four symptoms: erythema/haemorrhage, scarring/dryness, oedema, and excoriation/erosion. The sum of the individual scores indicated the clinical severity, which ranged from 0 to 12. The ear thickness was measured using a thickness gauge (Mitutoyo Corporation, Tokyo, Japan). To quantify pruritus symptoms in each mouse, we measured and recorded the frequency of dorsal skin rubbing with front or hind paws during a 10 min period immediately after DNCB sensitisation. The severity of skin lesions and scratching behaviour was assessed by two independent investigators blinded to the animal grouping. The results were calculated as the average of the two measurements.

### 2.4. Histopathological Observations

To evaluate skin thickening and cellular infiltration, the skin samples were fixed with 4% paraformaldehyde and embedded in paraffin. Thereafter, 5 *μ*m thick skin sections were cut and stained with hematoxylin and eosin (H&E) to detect inflammatory cells or with toluidine blue (TB) to detect mast cells. All images were observed under an optical microscope at 200x magnification for quantitative analysis.

### 2.5. Assessment of Skin Barrier Function

Dermalab® Series SkinLab Combo (Cortex Technology, Denmark) was used to evaluate shaved dorsal skin. Transepidermal water loss (TEWL) and hydration levels of the dorsal skin were measured.

### 2.6. Organ Index Analysis

On day 34 after dissection, the heart, liver, lungs, kidneys, spleen, and thymus were harvested and weighed using an electronic analytical balance to calculate the organ indices (organ weight/body weight, 100 × g/g).

### 2.7. Measurements of Serum Immunoglobulins (Ig) and Cytokines

Blood samples from each mouse were collected in coagulation-promoting tubes. The serum was separated from the blood by centrifugation (3000 × *g*, 4°C, 10 min) and stored at −80°C until use. The serum levels of IgE, IgG1, IgG2a, tumor necrosis factor- (TNF-) *α*, interleukin- (IL-) 4, IL-17A, and thymic stromal lymphopoietin (TSLP) were measured using mouse ELISA kits in accordance with the manufacturer's instructions.

### 2.8. RNA Isolation and Quantitative Real-Time Polymerase Chain Reaction (qRT-PCR) Analysis

Total RNA was extracted from dorsal skin samples using the TRIzol reagent. Equivalent amounts of RNA were reverse transcribed using the PrimeScript RT Reagent Kit (Takara, Tokyo, Japan). Thereafter, qRT-PCR was performed by amplifying cDNA prepared from the isolated RNA with SYBR Premix Ex Taq (Takara, Tokyo, Japan) using an ABI 7500 Sequence Detection system. *β*-Actin was used as an internal control. The amount of mRNA relative to the internal control was calculated using the equation 2^−△△CT^. The primer sequences used are listed in Table 1.

### 2.9. Statistical Analysis

The experimental data are expressed as the mean ± standard error of the mean. Statistical analyses were conducted using the GraphPad Prism software (GraphPad Software, San Diego, CA, USA). Mean values were compared using one-way analysis of variance followed by Tukey's multiple comparison test. Statistical significance was set at *P* < 0.05.

## 3. Results

### 3.1. SHED Characterisation

The SHEDs were isolated from pulp tissues of naturally exfoliated deciduous teeth of children aged 6–8 years. The cells from passage 4 were used in this study (Figure S1). The expression levels of stem cell markers of the SHEDs were analysed by flow cytometry. The SHEDs were positive for CD29, CD44, CD73, CD90, CD105, and CD146 and were negative for CD34 and CD45 (Figure S2).

### 3.2. SHEDs Attenuated DNCB-Induced AD-Like Clinical Manifestations and Scratching Behaviour

We injected the mice intravenously or subcutaneously with SHEDs in the elicitation phase to investigate the therapeutic potential of SHEDs. The experimental procedure is summarised in Figure 1(a). Compared with the control group, all DNCB-treated mice developed AD-like lesions, including erythema/haemorrhage, scarring/dryness, oedema, and excoriation/erosion (Figure 1(b)). Skin lesions were ameliorated in SHED-treated mice compared with those in the model group (Figure 1(b)).

The AD severity index was introduced to quantify the severity of skin lesions. The index was significantly higher in the model group than in the control group (Figure 1(c)). Additionally, the index was markedly decreased in the SHED-IV and SHED-SC groups (Figure 1(c)).

The difference in ear thickness between the left and right ears was calculated to evaluate the therapeutic effect of SHEDs in the mouse model. Notably, the difference in ear thickness was significantly higher in the model group than in the control group (Figure 1(d)). Moreover, the difference in ear thickness of the SHED-IV and SHED-SC groups was significantly lower than that in the model group (Figure 1(d)).

Intensive pruritus is the representative clinical manifestation of AD; therefore, we measured and recorded the scratching behaviour following DNCB treatment. The application of DNCB significantly increased the frequency of scratching episodes in the model group compared with that in the control group (Figure 1(e)). Notably, the frequency of scratching episodes was markedly decreased in the SHED-IV and SHED-SC groups (Figure 1(e)).

### 3.3. SHEDs Inhibited Epidermal Thickening and Mast Cell Infiltration

**Figure 2:**
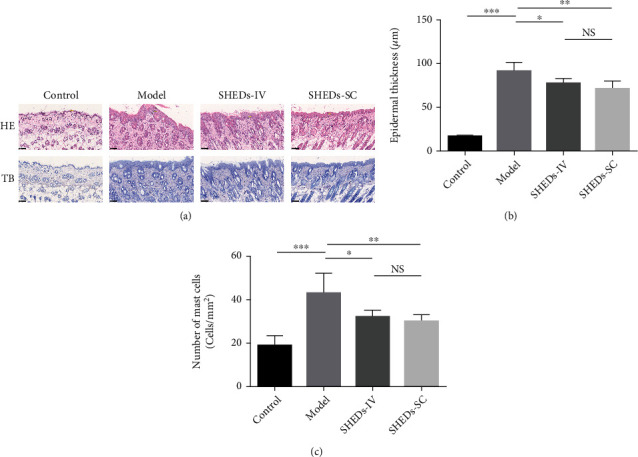
Effect of SHEDs on cutaneous histopathological observations. (a) Representative histologic findings of cutaneous tissue sections stained with hematoxylin and eosin (HE) or toluidine blue (TB) (magnification 200x; scale bar = 100 *μ*m). (b) The thicknesses of the epidermis in BALB/c mice of each group. (c) The number of infiltrated mast cells in 1 mm^2^ of skin lesion in BALB/c mice of each group. All the data are expressed as mean ± SD (*n* = 6). Three repeated times of each experiment were conducted.

We analysed the effect of SHEDs on the pathological changes in the skin lesions under an optical microscope (Figure 2(a)). Epidermal thickness was maintained at low levels in the control group, whereas DNCB administration significantly increased the epidermal thickness in the model group (Figure 2(b)). The epidermal thickness in the SHED-IV and SHED-SC groups markedly decreased compared to that in the control group (Figure 2(b)).

Furthermore, TB was used to alter the infiltration of mast cells into the skin (Figure 2(a)). The total mast cell counts were significantly higher in the model group than in the control group (Figure 2(c)). Moreover, mast cell infiltration was significantly ameliorated in the SHED-IV and SHED-SC groups (Figure 2(c)).

### 3.4. SHEDs Restored DNCB-Induced Defects in Skin Barrier Function

**Figure 3:**
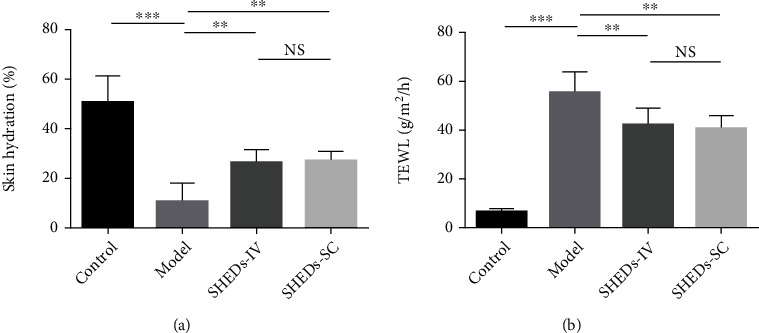
Effect of SHEDs on skin barrier. (a) Skin hydration in BALB/c mice of each group. (b) Transepidermal water loss (TEWL) in BALB/c mice of each group. All the data are expressed as mean ± SD (*n* = 6). Three repeated times of each experiment were conducted.

Skin barrier function was evaluated using TEWL and skin hydration measurements. DNCB application promoted an increase in TEWL and a decrease in skin hydration levels in the model group compared with those in the control group (Figure 3(a)). Significant differences were also observed between the SHED-IV and SHED-SC groups and the model group. SHED treatment increased skin hydration levels and reduced TEWL (Figure 3(b)).

### 3.5. SHEDs Regulated the Serum Levels of Igs and Cytokines

**Figure 4:**
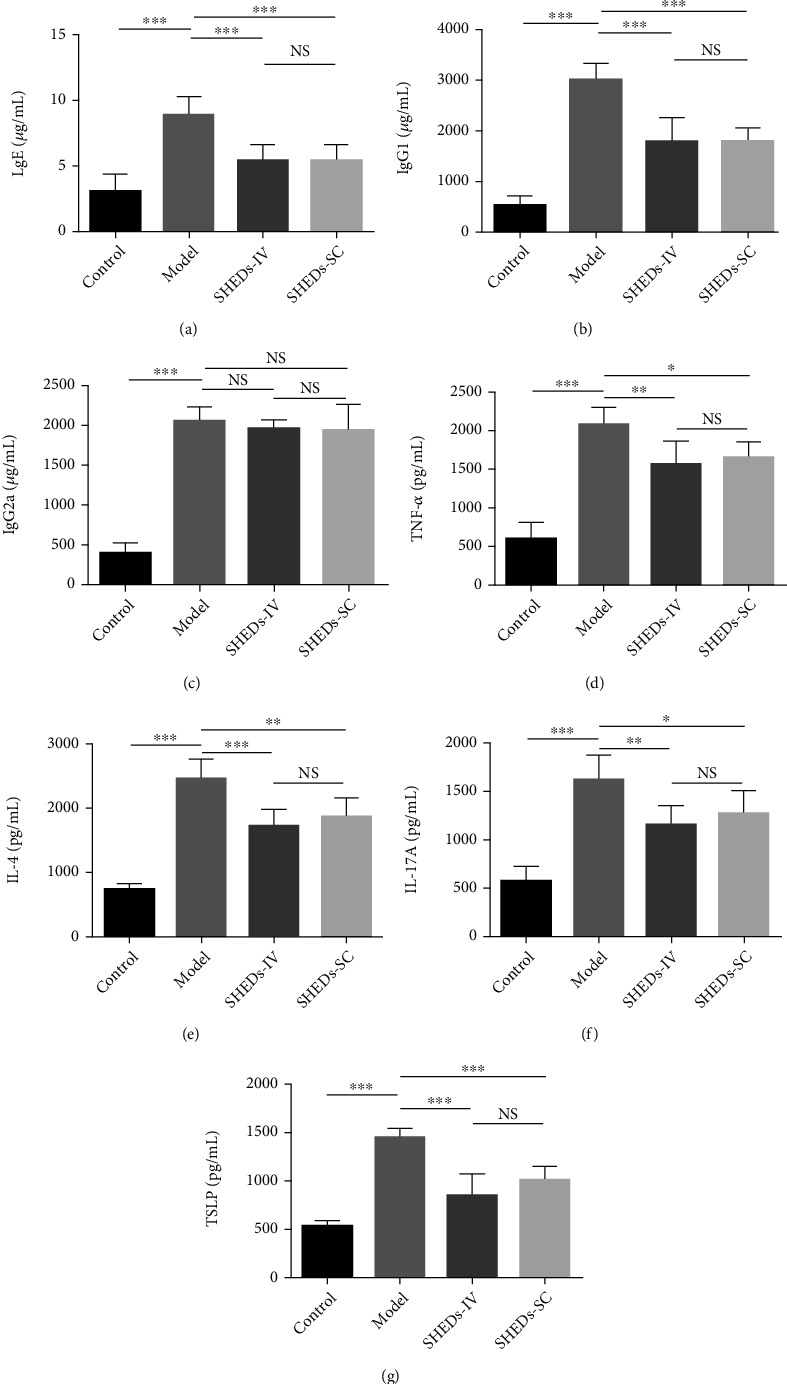
Effect of SHEDs on AD-related immunoglobulin and cytokine expression in serum and skin tissue. (a–c) The immunoglobulin levels of IgE, IgG1, and IgG2a in serum. (d–g) The cytokine levels of TNF-*α*, IL-4, IL-17A, and TSLP in serum. All the data are expressed as mean ± SD (*n* = 6). Three repeated times of each experiment were conducted.

The serum levels of IgE, IgG1, and IgG2a were significantly increased in the model group compared with those in the control group (Figures 4(a)–4(c)). Furthermore, SHED administration significantly reduced the serum levels of IgE and IgG1 in the SHED-IV and SHED-SC groups compared with those in the model group (Figures 4(a) and 4(b)). However, no difference in serum IgG2a levels was observed between the groups (Figure 4(c)).

The serum levels of serum cytokines such as TNF-*α*, IL-4, IL-17A, and TSLP in the model group were significantly higher than those in the control group (Figures 4(d)–4(g)). This upregulation was alleviated by SHED administration in the SHED-IV and SHED-SC groups (Figures 4(a)–4(g)).

### 3.6. SHEDs Reduced DNCB-Induced Spleen Enlargement

No remarkable differences in the organ indices of the heart, kidneys, and lungs were observed between the four groups (Table 2). However, the organ indices of the liver and spleen in the model group were significantly increased compared with those in the control group (Table 2), and the enlarged spleens were significantly reduced in size in the SHED-IV group (Table 2). The organ indices of the thymus in the model group were significantly lower than those in the control group (Table 2). Notably, SHED administration did not restore normal thymus size in the SHED-IV and SHED-SC groups (Table 2).

### 3.7. SHEDs Regulated DNCB-Induced Cytokine Gene Expression in Dorsal Skin

**Figure 5:**
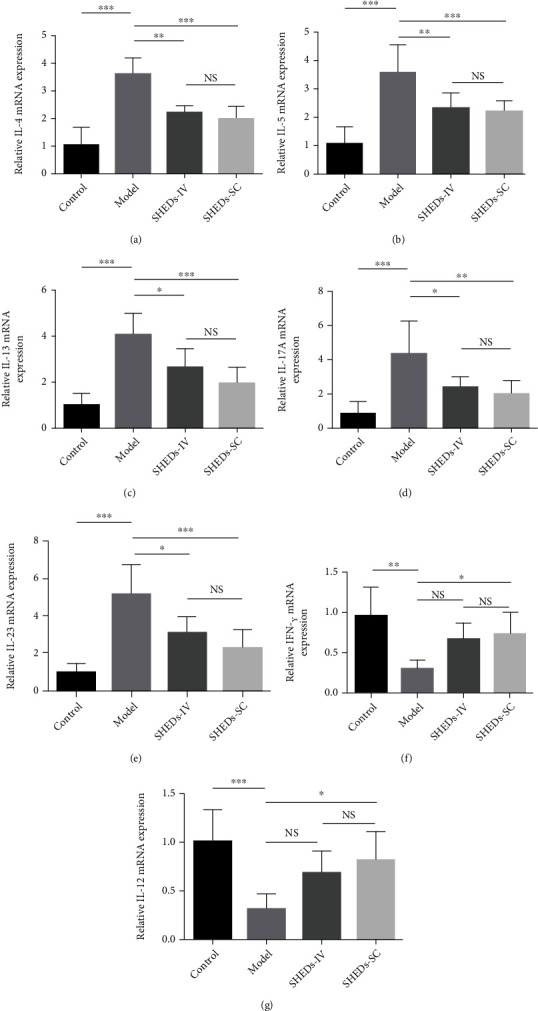
Effect of SHEDs on AD-related cytokine expression in skin tissue. (a–e) The relative mRNA levels of Th2- and Th17-mediated cytokines including IL-4, IL-5, IL-13, IL-17A, and IL-23. (f and g) The relative mRNA levels of Th1-mediated cytokines including IFN-*γ* and IL-12. All the data are expressed as mean ± SD (*n* = 6). Three repeated times of each experiment were conducted.

To understand the mechanism of SHEDs in alleviating AD symptoms, we examined the expression levels of AD-related inflammatory cytokines in skin lesions. Compared with those in the control group, DNCB markedly increased the levels of T helper (Th)2- and Th17-mediated cytokines, including IL-4, IL-5, IL-13, IL-17A, and IL-23, in the model group. In contrast, SHED administration downregulated the expression of the AD-related cytokine genes (Figures 5(a)–5(e)). Moreover, the expression of Th1-mediated cytokines, including interferon- (IFN-) *γ* and IL-12, was markedly downregulated in the model group; however, SHED administration restored the expression of the cytokine genes (Figure 5(a) and 5(g)).

## 4. Discussion

Atopic dermatitis is an inflammatory skin disease caused by immune dysregulation and complex interactions between genetic predispositions and environmental factors [1]. Current AD treatments include the use of antihistamines, steroids, and immune suppressants, which are associated with limited therapeutic efficacy and side effects owing to their long-term usage [16]. Therefore, the exploration and development of novel, safer, and more effective AD treatment strategies are urgently needed. Recently, MSCs have shown promise as potential cell-based therapies for AD [10]. Studies have demonstrated that MSCs can suppress the allergic reaction in an AD mouse model [17–19]. Moreover, several clinical trials are investigating the benefits of MSCs in AD treatment [10, 20]. In this study, we established an AD mouse model by repeated percutaneous administration of DNCB and investigated the potential therapeutic effects of SHEDs in the model.

Notably, we found that SHED administration attenuated DNCB-induced AD lesions and symptoms; it considerably reduced the severity of skin lesions, including the dermatitis score and ear thickness. Pruritus is a representative clinical feature of AD and can initiate a vicious itch-scratch cycle. Therefore, controlling pruritus is of vital importance in AD treatment [21]. SHED administration also reduced scratching behaviour in the AD model. Moreover, epidermal hyperplasia is a typical histopathological change that occurs in AD skin lesions. Intriguingly, histological analysis indicated that SHED treatment reduced epidermal thickness. Mast cells are the major effector cells in immediate hypersensitivity that are activated via the high-affinity IgE receptor Fc*ϵ*RI. Studies have revealed that mast cells play a role in the spontaneous or induced development of AD-like skin lesions [22–24]. Notably, we found that SHED treatment ameliorated mast cell infiltration in the dermis.

Patients with AD have a disrupted skin barrier. In particular, skin barrier-related proteins such as filaggrin, involucrin, and loricrin are present at low levels in patients with AD [25]. The typical manifestations of AD include dry skin and loss of skin hydration, which allow allergens or microbes to penetrate the skin and induce an immunological response. Therefore, restoring the skin barrier is crucial for preventing the development of AD. In this study, DNCB challenge led to loss of skin hydration and increased TEWL in the lesions. However, SHED-treated mice had significantly increased skin hydration and decreased TEWL, suggesting that SHEDs contributed to restoring the skin barrier.

Immunologic dysregulation is a major factor in the pathogenesis of AD with dominant Th2­skewed immune dysregulation. TSLP is an epithelial cell-derived cytokine, which is highly expressed in the keratinocytes of patients with AD. Furthermore, TSLP drives AD progression by activating dendritic cells and inducing Th2 responses [26]. Activated Th2 lymphocytes produce high levels of IL-4, IL-5, and IL-13 [27]. IL-4 and IL-13 promote isotype switching in B cells, which results in the production of antigen-specific IgE and IgG1 [27]. IgE binds to Fc*ϵ*RI on the surface of mast cells, which leads to the release of various inflammatory mediators, such as histamine, chemokines, and cytokines [28]. IL-5 plays a crucial role in eosinophil development, proliferation, and survival [29]. Additionally, upregulation of the Th2 response can inhibit the generation of Th1 cells, resulting in the deficiency of Th1-derived cytokines, including IFN-*γ* and IL-12 [30]. In an allergic rhinitis mouse model, SHEDs inhibited the Th2 immune response by downregulating IL-4, IL-5, and IL-13 levels in spleen lymphocytes and decreasing the production of serum IgE and IgG1 [14]. In this study, SHEDs reduced the mRNA levels of IL-4, IL-5, and IL-13 in the skin and the expression levels of IgE, IgG1, and TSLP in the serum of AD mice. Furthermore, SHEDs increased the mRNA levels of IFN-*γ* and IL-12 in the skin. These results suggested the role of SHEDs in normalising Th2-deviated immunologic dysregulation and restoring the Th1/Th2 balance.

In addition to the Th2 response, the Th17 response is dysregulated in AD. Th17 cell counts are increased in patients with AD; particularly, Th17 cells infiltrate the dermis of the lesion, and Th17-related cytokines, such as IL-17 and IL-23, are overexpressed. IL-17 can induce the expression of various proinflammatory cytokines, such as IL-6 and IL-8, which leads to increased T cell migration to the skin [31]. IL-17 also promotes neutrophil recruitment, which leads to neutrophil- and eosinophil-mediated inflammation in AD [32]. Notably, SHEDs significantly inhibit Th17 cells in vitro and can reverse systemic lupus erythematosus-associated disorders by downregulating Th17 cell responses in MRL/lpr mice [13]. SHEDs were shown to reduce IL-17A levels in the spleens of allergic rhinitis mice and downregulate IL-17A secretion from peripheral blood mononuclear cells derived from patients with allergic rhinitis [14]. In this study, SHED administration decreased the expression of IL-17A and IL-23 in the skin, indicating that SHEDs could inhibit the Th17 response in AD and delay disease development.

In recent years, BM-MSCs, UCB-MSCs, and AD-MSCs have been used in AD treatment [10]. Nevertheless, obtaining BM-MSCs and AD-MSCs requires an invasive extraction process, and UCB-MSCs must be collected at birth, which restricts their application. In contrast, SHEDs are easily obtained using noninvasive methods without risk of complications. Moreover, SHEDs have a higher proliferation ability than BM-MSCs and AD-MSCs [12]. They are usually obtained during childhood because AD typically develops in childhood. Therefore, autologous SHEDs are promising for AD treatment, particularly in paediatric patients.

In previous studies, stem cell administration routes and conditioned medium varied in patients with AD, as well as mouse models, from subcutaneous and intravenous injections to topical application [19, 33, 34]. Some studies have reported free distribution and homing effects of MSCs on damaged tissues [35]. Of note, Kim et al. reported that subcutaneous application of UCB-MSCs has a stronger therapeutic effect than intravenous administration in an AD mouse model [36]. In this study, SHEDs were administered intravenously or subcutaneously, and no significant differences were observed between these two routes.

This study had some limitations. We utilised SHEDs only in the DNCB-induced AD mouse model, which does not reflect patients with AD. Furthermore, the mechanism by which SHEDs regulate the immune balance and skin barrier function remains unknown. Future studies will be required to elucidate the detailed mechanism and evaluate various aspects of SHED therapy for AD.

## 5. Conclusions

In summary, our study demonstrated that SHEDs significantly ameliorated AD-like symptoms and repaired the dysfunctional skin barrier in DNCB-sensitised mice. SHEDs may have excellent immunomodulatory abilities and may be considered potential therapeutic agents for AD treatment.

## Figures and Tables

**Table 1:** Primer sequences for quantitative real-time polymerase chain reaction (qPCR).

Gene	Forward primer	Reverse primer
IL-4	GTCTGTAGGGCTTCCAAGGT	ATGATGCTCTTTAGGCTTTCCAG
IL-5	CCTCATCCTCTTCGTTGCATCAGG	TGATCCTCCTGCGTCCATCTGG
IL-12	GCAGAAAGGTGCGTTCCTCG	ATGTGCAGGTGTGGTTGGGC
IL-13	CCTGGCTCTTGCTTGCCT T	GGTCTTGTGTGATGTTGCTCA
IL-17A	TCAGCGTGTCCAAACACTGAG	CGCCAAGGGAGTTAAAGACTT
IL-23	ACCTGTAGTGGTGGTGGTGGAG	GGACCAGATAACTGTTGGCAGAGC
IFN-*γ*	GCCACGGCACAGTCATTGA	TGCTGATGGCCTGATTGTCTT
GADPH	GTCAAGGCCGAGAATGGGAA	CTCGTGGTTCACACCCATCA

**Table 2:** Effect of SHED administration on organ indexes (100∗g per g body weight).

Group	Organ index (100∗g/g body weight)					
	Heart	Liver	Spleen	Lung	Kidney	Thymus
Control	0.58 ± 0.01	4.69 ± 0.18	0.38 ± 0.02	0.70 ± 0.07	1.52 ± 0.08	0.21 ± 0.03
Model	0.58 ± 0.03	5.26 ± 0.33^#^	0.75 ± 0.10^###^	0.68 ± 0.03	1.55 ± 0.08	0.13 ± 0.03^###^
SHEDs-IV	0.54 ± 0.06	5.34 ± 0.32	0.63 ± 0.08^∗^	0.73 ± 0.04	1.55 ± 0.05	0.11 ± 0.02
SHEDs-SC	0.52 ± 0.03	5.52 ± 0.45	0.69 ± 0.05	0.65 ± 0.07	1.59 ± 0.11	0.12 ± 0.03

Note: all the data are expressed as mean ± SD (*n* = 6). ^#^*P* < 0.05 and ^###^*P* < 0.001 vs. control group; ^∗^*P* < 0.05 vs. model group.

## Data Availability

All primary datasets generated for this study are available from the corresponding author upon request.

## Supplementary Materials

Supplementary Materials Figure S1: the morphology of stem cells from human exfoliated deciduous teeth (passage 4). Figure S2: the expression of stem cell markers assessed via flow cytometry. The positive rate of CD34 and CD45 was less than 2%. The positive rate of CD29, CD44, CD73, and CD90 was more than 95%. The positive rate of CD105 was more than 90%. The positive rate of CD146.
