# Supplementary material for: Stem Cells from Human Exfoliated Deciduous Teeth Attenuate Atopic Dermatitis Symptoms in Mice through Modulating Immune Balance and Skin Barrier Function
Source: Mediators Inflamm. 2022 Jul 21;2022:6206883. doi: 10.1155/2022/6206883 (PMC9334056; doi:10.1155/2022/6206883)
Supplement: Supplementary Materials — Figure S1: the morphology of stem cells from human exfoliated deciduous teeth (passage 4). Figure S2: the expression of stem cell markers assessed via flow cytometry. The positive rate of CD34 and CD45 was less than 2%. The positive rate of CD29, CD44, CD73, and CD90 was more than 95%. The positive rate of CD105 was more than 90%. The positive rate of CD146. [file 6206883.f1.docx]

Supplementary Material


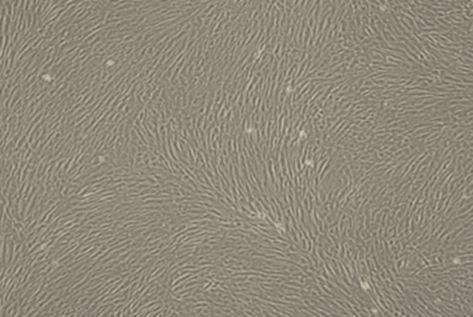


Figure S1. The morphology of stem cells from human exfoliated deciduous teeth (passage 4).


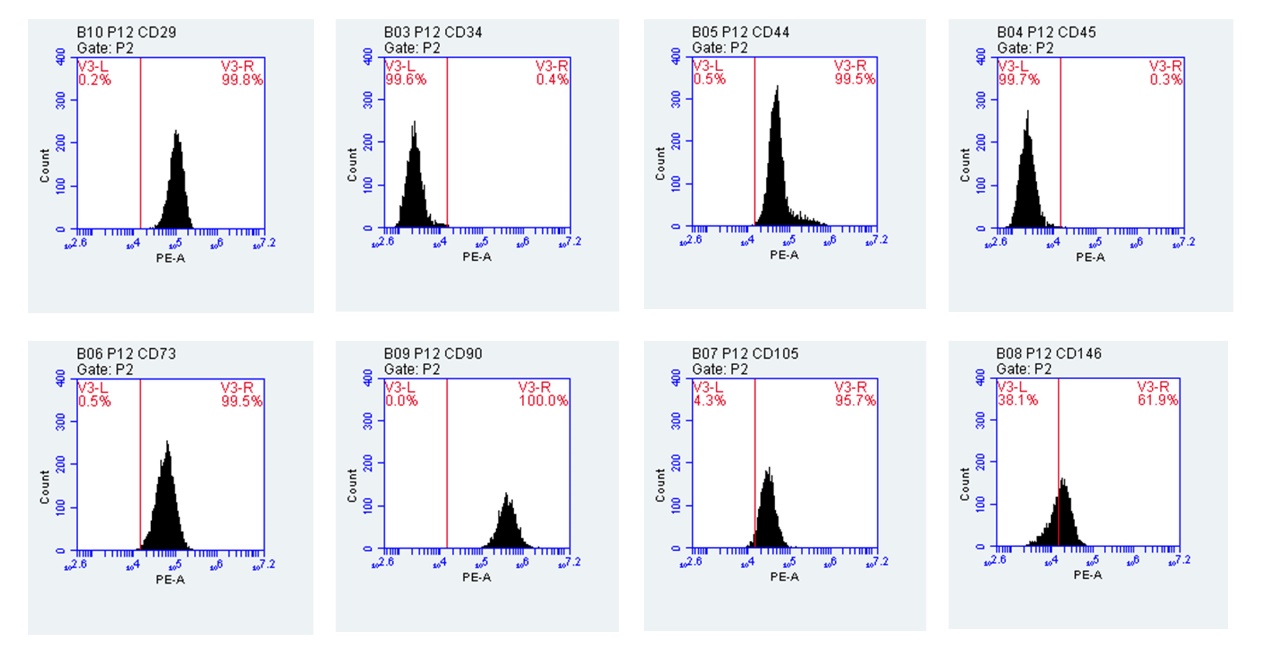


Figure S2. The expression of stem cell markers assessed via flow cytometry. The positive rate of CD34 and CD45 was less than 2%. The positive rate of CD29, CD44, CD73 and CD90 was more than 95%. The positive rate of CD105 was more than 90%. The positive rate of CD146 was more than 60%.
